# Hereditary hemorrhagic telangiectasia with liver cirrhosis: a case report

**DOI:** 10.1186/s12876-021-01913-3

**Published:** 2021-09-06

**Authors:** Linxia Xu, Feng Xu, Qizhi Wang, Xiquan Ke

**Affiliations:** Department of Gastroenterology, The First Affiliated Hospital of Bengbu Medical College, Bengbu, 233004 Anhui China

**Keywords:** Hereditary hemorrhagic telangiectasia, Liver cirrhosis, Thalidomide, Argon plasma coagulation, Case report

## Abstract

**Background:**

Hereditary hemorrhagic telangiectasia is an autosomal dominant hereditary hemorrhagic disease. Its main feature is an abnormal structure of the blood vessel wall. Cirrhosis of the liver is a common chronic progressive disease with one or more causes in which diffuse liver damage occurs after long-term or repeated injury. Liver cirrhosis can cause dilation of gastrointestinal capillaries. Many patients with hereditary hemorrhagic telangiectasia accompanied by gastrointestinal vascular malformations and liver cirrhosis may be diagnosed only with liver cirrhosis if the clinician does not pay attention to physical examination findings and family history. Moreover, general treatment measures, such as blood transfusion, iron supplementation, and application of hemostatic drugs, are less effective for bleeding in patients with hereditary hemorrhagic telangiectasia than in those with liver cirrhosis alone.

**Case presentation:**

Here, we report the rare case of a 75-year-old Chinese man who was admitted to the hospital with repeated melena and epistaxis. He was diagnosed with unexplained liver cirrhosis, which was later confirmed as hereditary hemorrhagic telangiectasia. Subsequently, we implemented the treatment intervention of oral thalidomide combined with gastrointestinal argon plasma coagulation. A follow-up of more than 8 months showed that the treatment effect was excellent.

**Conclusions:**

If patients with liver cirrhosis and gastrointestinal vascular malformations also have a family history of epistaxis, special attention should be paid to targeted physical examination results, and the possibility of hereditary hemorrhagic telangiectasia should be considered. Moreover, for patients with hereditary hemorrhagic telangiectasia and both gastrointestinal bleeding caused by gastrointestinal capillaries and repeated epistaxis, when other general treatment measures are ineffective, thalidomide combined with gastrointestinal argon plasma coagulation may be an effective intervention.

## Background

Hereditary hemorrhagic telangiectasia (HHT) is an autosomal dominant hereditary hemorrhagic disease characterized by skin and/or mucosal telangiectasia and arteriovenous malformations (AVMs), with a global prevalence of at least 1/5000 [1]. The occurrence of HHT is closely related to gene mutations; of those with a pathogenic mutation, 61% have ENG mutations, 37% have ACVRL1 mutations, and 2% have MADH4 mutations [2]. HHT can occur in childhood, and bleeding symptoms gradually increase with age.

Vascular endothelial growth factor (VEGF) is a key mediator in the early stages of angiogenesis and contributes to the formation of primitive endothelial blood vessels. Studies have shown that HHT is an angiogenic disease, and the overexpression of VEGF is related to its occurrence [3]. The deformed blood vessels are thin and fragile, lack smooth muscle cells, rupture easily, and often cause repeated and refractory bleeding from the involved blood vessels [4], with nose bleeding being the most common manifestation. Telangiectasias can occur in the gastrointestinal tract, liver, lungs, fundus of the eyes, brain, and other organs, which can cause hemorrhages in the corresponding part. In severe cases, cerebral hemorrhage can occur [5]. Current international guidelines for HHT recommend annual measurement of hemoglobin and serum iron levels beginning when the patient is 35 years of age and suggest that endoscopic evaluation should be performed in the case of anemia that is disproportionate to the amount of epistaxis [1].

Currently, the diagnosis of HHT is based on mutant gene detection and the clinical diagnostic criteria established by the Scientific Advisory Committee of the International HHT Fund in 2000 [6]: first, epistaxis, or spontaneous, recurrent nose bleeds; second, telangiectasias, including multiple telangiectasias in the lips, oral cavity, fingers, and nose; third, visceral lesions, including gastrointestinal telangiectasia (with or without bleeding), pulmonary AVM, hepatic AVM, cerebral AVM, and spinal AVM; fourth, family history, including a first-degree relative with HHT according to these criteria. Meeting three or more of the above four items is the diagnosis of the disease, having two of the above items is suspicious, and having less than two items can rule out HHT.

## Case presentation

**Fig. 1:**
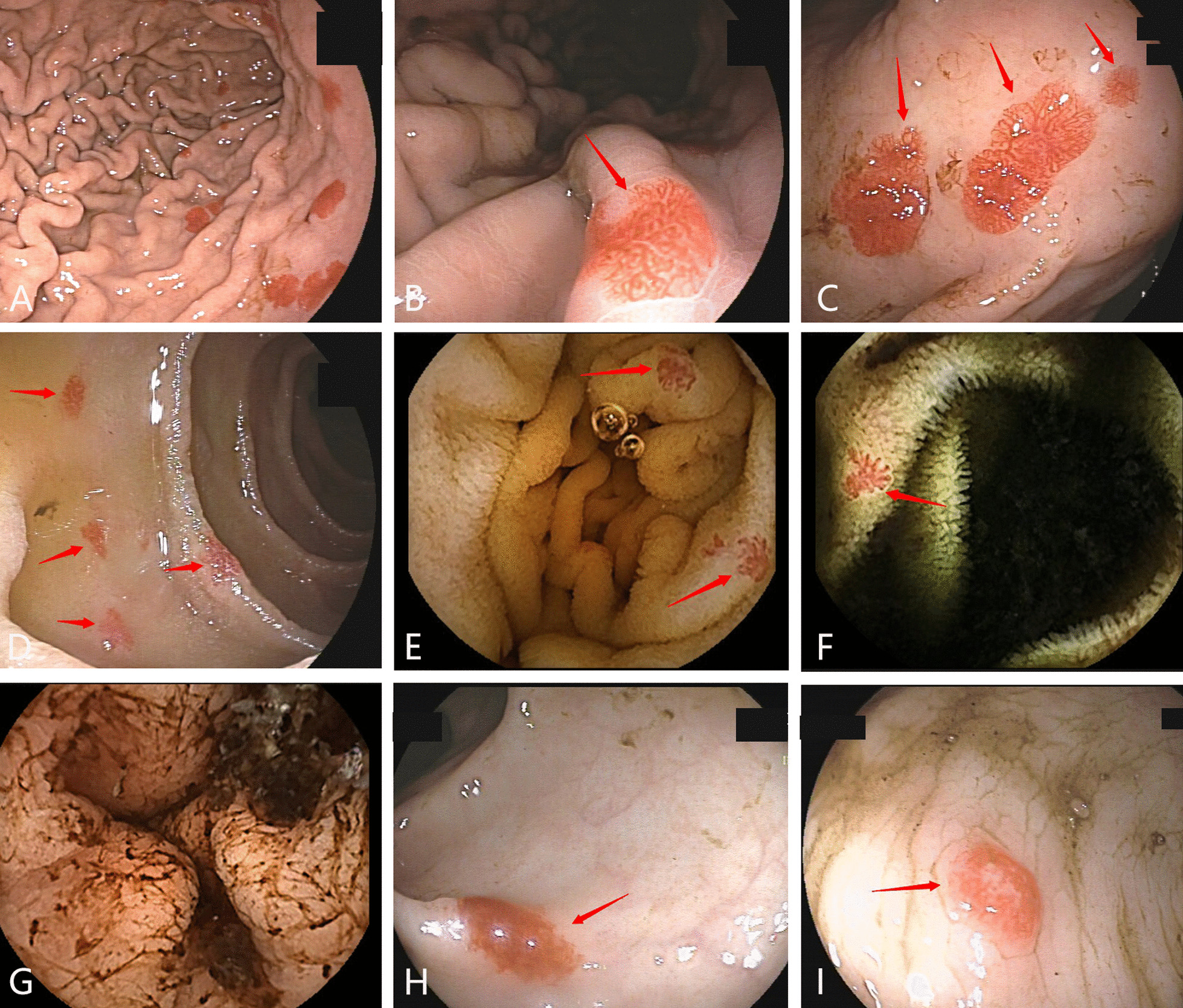
Multiple telangiectasias in the stomach, duodenum, jejunum, ileum, and colon viewed under endoscopy. Multiple telangiectasias in the stomach (**A–C**) and duodenum (**D**) can be seen under gastroscopy. **E, F** Multiple telangiectasias in the jejunum and ileum can be seen under capsule endoscopy. **G** An old hemorrhage in the intestine can be seen under capsule endoscopy. **H, I** Multiple telangiectasias in the colon can be seen under colonoscopy

**Fig. 2:**
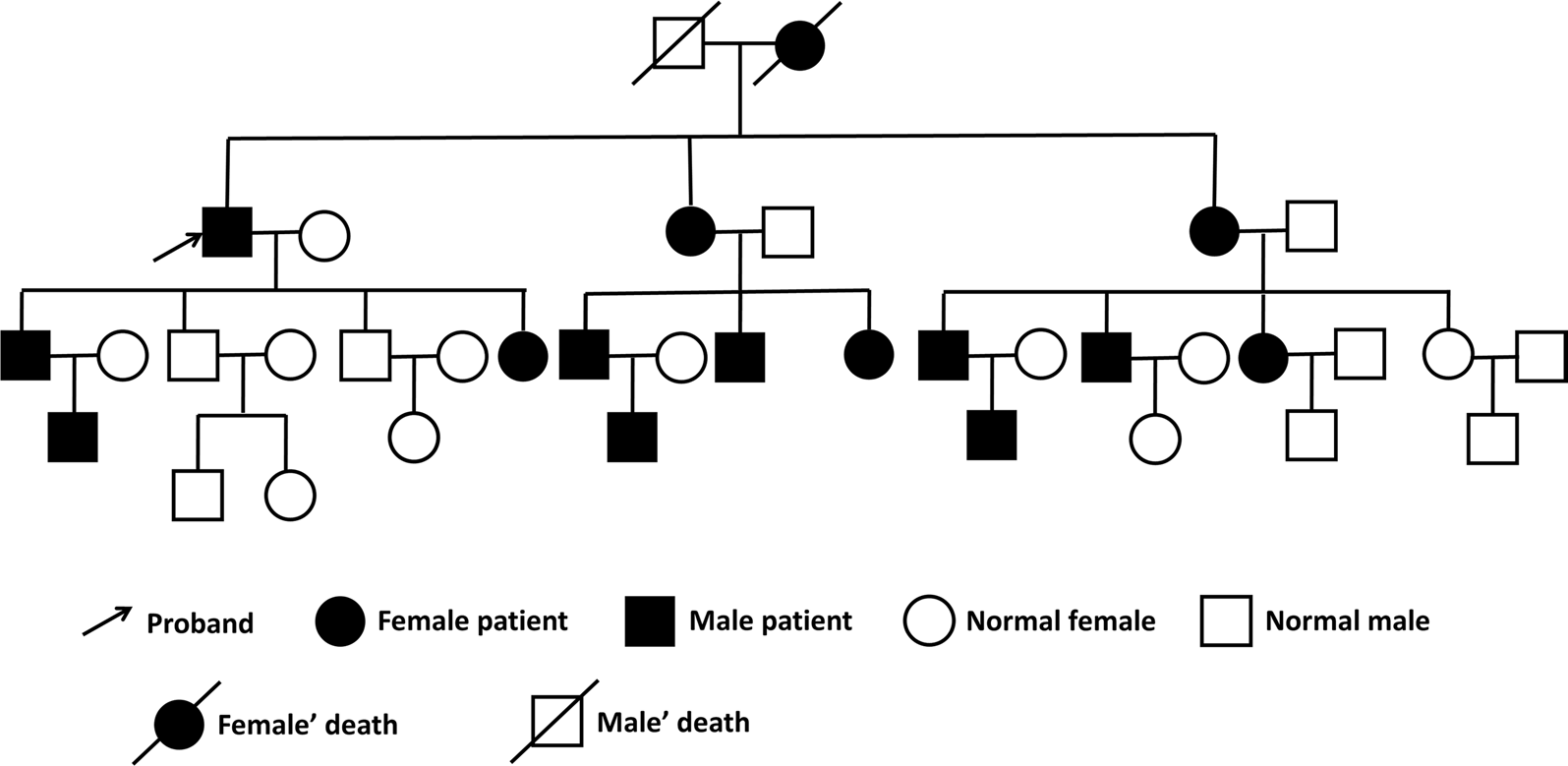
Pedigree chart of epistaxis. The patient's mother and two younger sisters, as well as multiple of their offspring, all have the same symptoms as the patient, manifested by repeated spontaneous epistaxis that began in childhood

**Fig. 3:**
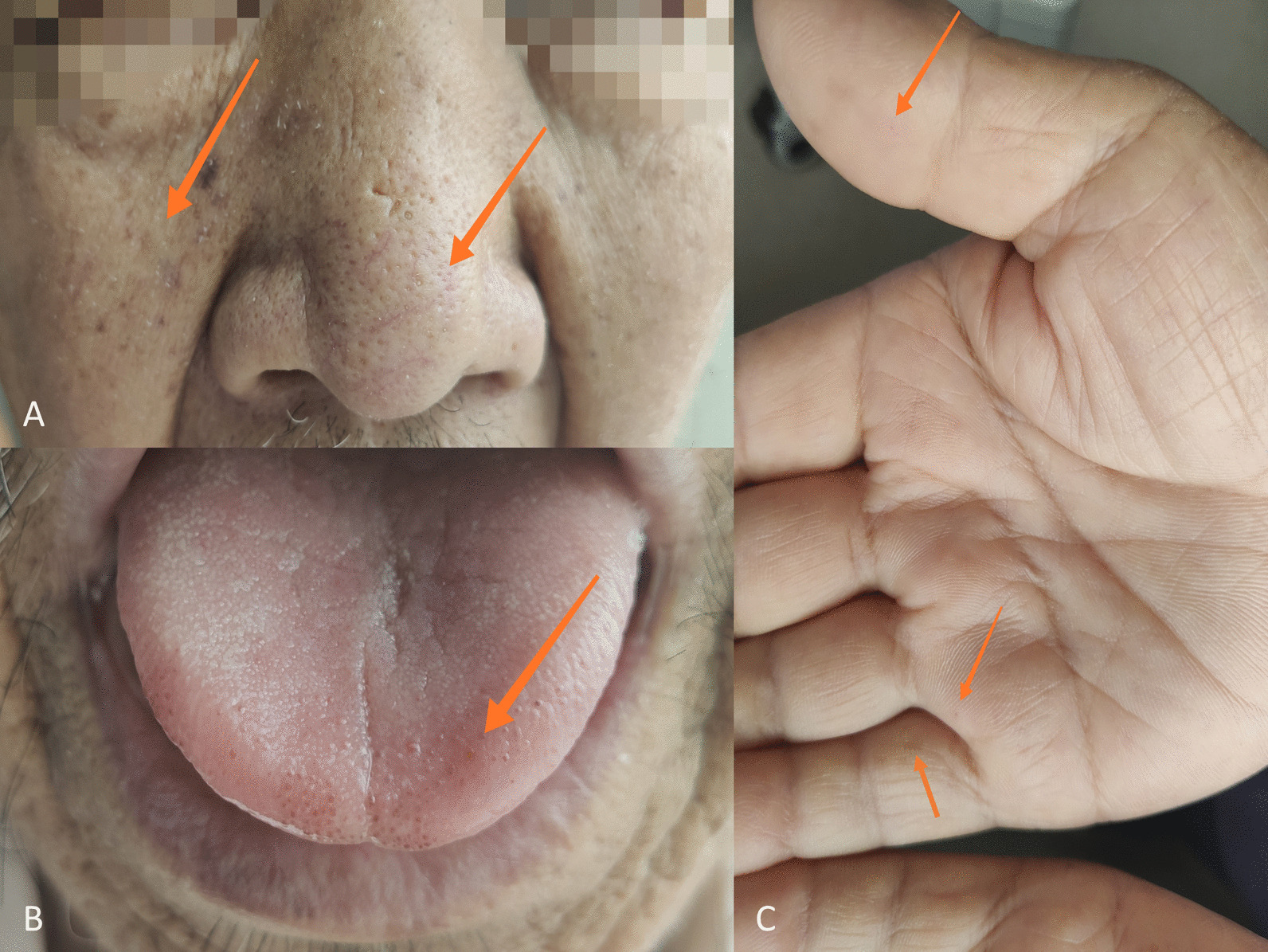
Nose, cheek, tongue mucosa, and hand telangiectasias. The physical examination showed multiple telangiectasias in the patient’s nose, cheeks, tongue mucosa, and hand

A 75-year-old man who had experienced melena and anemia for more than 20 years was admitted to the Gastroenterology Department of our hospital. Physical examination showed that the patient had mild spleen enlargement, and mild edema of both lower limbs. Ultrasonography revealed cirrhosis, mild spleen enlargement and a small amount of ascites and ruled out Budd-Chiari syndrome. The patient had no long-term history of heavy alcohol consumption. He had no history of hypertension, diabetes, coronary heart disease, or hepatitis. No abnormalities were observed in autoimmune liver disease-related indicators. The patient was admitted to the hospital with a hemoglobin level of 41 g/L, mean corpuscular volume of 69.6 fl, and ferritin concentration of 10.00 ng/mL (normal range 13–150 ng/mL), low albumin level (32 g/L), and slightly elevated liver transaminase (ALT 60U/L). There were no esophageal/gastric varices and portal hypertensive gastropathy under gastroscopy. Ordinary endoscopy and capsule endoscopy revealed multiple telangiectasias in the stomach, duodenum, small intestine, and colon, with a predominance in the fundus of the stomach, the body of the stomach, the duodenum, and the proximal jejunum, totaling approximately 30 (Fig. 1). Cirrhosis of the liver can cause gastrointestinal vascular malformations, and he was diagnosed with unexplained liver cirrhosis. The patient was treated with hemostasis, blood transfusion, iron supplementation, liver protection, and other treatments after admission, but after repeated blood tests, his hemoglobin levels did not increase significantly. During hospitalization, the patient had recurrent melena and nosebleeds and said that he had experienced nosebleeds repeatedly since childhood. Several close relatives in the family also had epistaxis similar to that of the patient (Fig. 2). On physical examination, the patient’s nose, cheeks, tongue tip mucosa, and hands had multiple telangiectasias (Fig. 3). The patient refused to be tested for mutations, but according to the criteria of the Scientific Advisory Committee of the International HHT Fund in 2000 [6], the diagnosis of HHT in this patient was clear. In this case, the condition affected the entire intestinal tract; thus, it was classified as Moore type III [7], which is relatively rare.

**Fig. 4:**
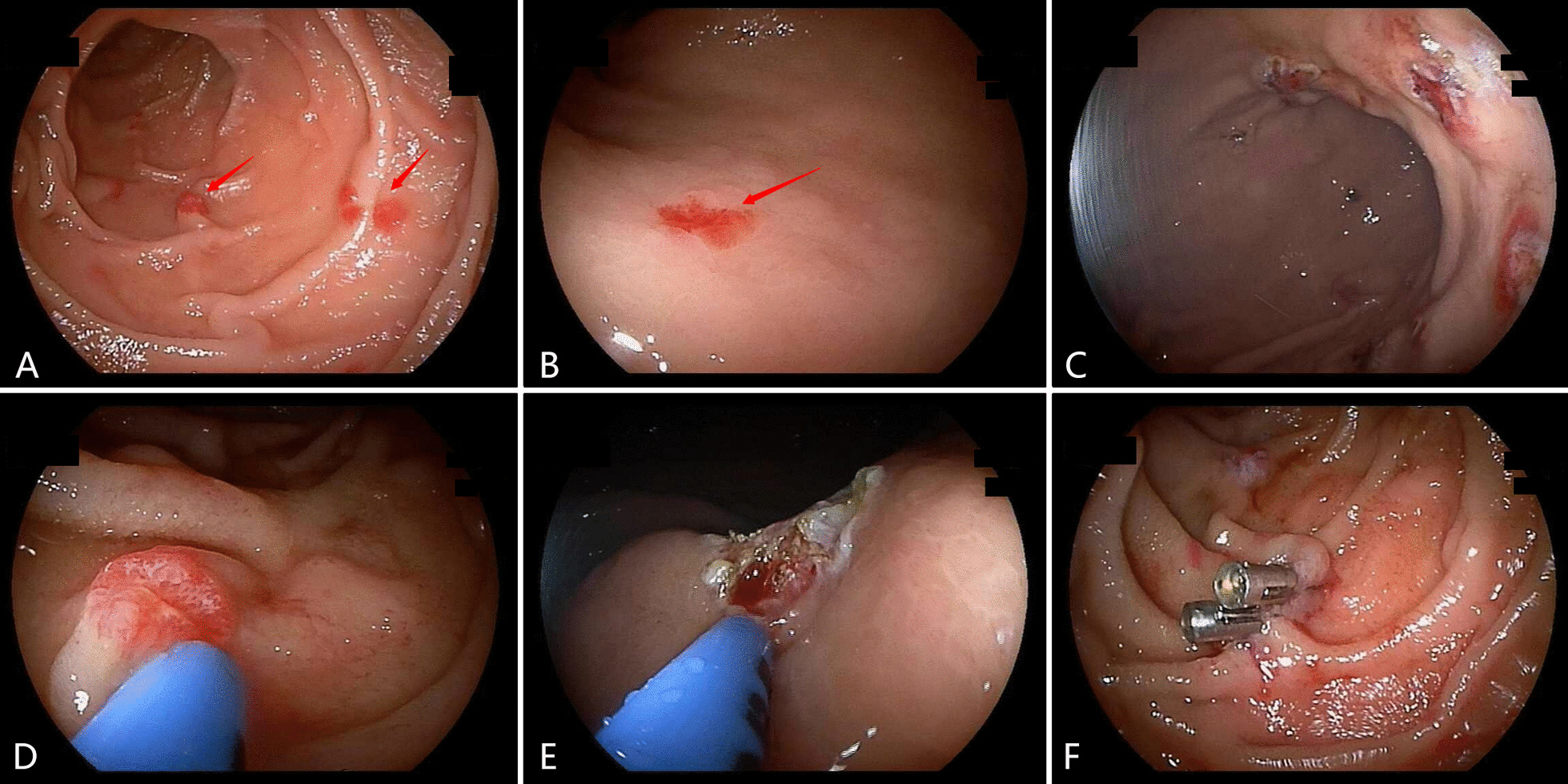
Observations of treatment under double-balloon endoscopy. **A, B** Multiple telangiectasias can be seen under double-balloon endoscopy. **C** Under double-balloon endoscopy, argon plasma coagulation can be seen being used for endoscopic treatment. After treatment, angiodysplasia had completely disappeared with mucosal carbonization. **D–F** Individual areas with abundant blood supply have larger wounds after electrocoagulation, and titanium clips were used to close the wounds

General treatment measures, such as blood transfusion and hemostasis, are ineffective in HHT. During hospitalization, the patient was administered oral thalidomide 50 mg twice a day, and argon plasma coagulation (APC) under double-balloon endoscopy was performed at the same time (Fig. 4). Eight months after being discharged from the hospital, the patient was taking oral thalidomide 50 mg twice a day as a maintenance dose, with no drug-related side effects. The patient’s fatigue symptoms were significantly reduced compared with before treatment, and a recheck of his hemoglobin showed that it was significantly higher than it was before treatment. He underwent regular review after discharge. At the latest review, the patient's weight increased by 3 kg compared to before the first admission. No abnormal liver function and no ascites occurred more than 8 months after discharge. Additionally, the patient had no more melena, nose bleeding, or bleeding from other organs. The patient is still being followed up.

## Discussion and conclusions

In our case, the elderly man met at least three diagnostic criteria: (1) spontaneous, recurrent nose bleeds, (2) multiple telangiectasias of the skin and mucous membranes, and (3) gastrointestinal telangiectasia. Thus, the patient clearly had HHT. Moreover, he had a family history of epistaxis and was the first patient in his family to be diagnosed with HHT. The diagnosis of this patient may provide guidance for the treatment of nose bleeds in other members of his family.

APC has been widely used in the treatment of gastrointestinal bleeding and vascular malformations [8] and can be used in AVMs related to HHT. Studies have found that thalidomide is involved in reducing local concentrations of VEGF in malformed blood vessels [9, 10] and is known for its ability to inhibit the production of abnormal blood vessels, stimulate parietal cell coverage, repair vascular wall defects, and stimulate vessel maturation [11]. Some randomized controlled trials have verified that thalidomide is an effective and relatively safe treatment for patients with refractory bleeding from gastrointestinal vascular malformations [10].

Teratogenicity is the most serious side effect of thalidomide [12], which is absolutely contraindicated in women who are or could become pregnant. Moreover, evaluation of prothrombotic conditions should be considered before treatment with thalidomide is started. Patients at high risk of thromboembolic events should be excluded from this treatment [13]. The most commonly reported adverse effects of thalidomide are peripheral neuropathy, drowsiness, and dizziness [13]. The symptoms can be improved after symptomatic treatment.

For patients with liver cirrhosis and gastrointestinal vascular malformations, physicians should also focus on other factors in addition to the history of liver cirrhosis. In particular, when a patient has a clear family history of epistaxis or severe anemia that cannot be explained by simple nose bleeds, physicians should pay close attention to targeted physical examination results and family history information and consider the possibility of HHT as a diagnosis. Intestinal and hepatic involvement can negatively affect the progression of the disease; therefore, we believe that in the near future, it will be important to screen all patients with HHT for gastrointestinal manifestations, especially those with severe anemia that is not proportional to the degree of nose bleeds.

Thalidomide has a low toxicity and safe preparation. For patients with HHT and gastrointestinal bleeding caused by gastrointestinal telangiectasia and repeated epistaxis, when other general treatment measures are ineffective, the treatment plan of thalidomide combined with APC may be effective.

## Data Availability

The datasets used and/or analyzed during the current case report are available from the corresponding author upon reasonable request.

## Abbreviations

APC: Argon plasma coagulation
AVM: Arteriovenous malformation
HHT: Hereditary hemorrhagic telangiectasia
VEGF: Vascular endothelial growth factor
